# Inequalities in mortality of men by oral and pharyngeal cancer in Barcelona, Spain and São Paulo, Brazil, 1995–2003

**DOI:** 10.1186/1475-9276-7-14

**Published:** 2008-06-04

**Authors:** José Leopoldo Ferreira Antunes, Carme Borrell, Glòria Pérez, Antonio Fernando Boing, Victor Wünsch-Filho

**Affiliations:** 1 School of Dentistry, University of São Paulo, São Paulo, Brazil; 2 Agència de Salut Pública de Barcelona, Barcelona, Spain; 3 School of Public Health, University of São Paulo, Brazil

## Abstract

**Background:**

Large inequalities of mortality by most cancers in general, by mouth and pharynx cancer in particular, have been associated to behaviour and geopolitical factors. The assessment of socioeconomic covariates of cancer mortality may be relevant to a full comprehension of distal determinants of the disease, and to appraise opportune interventions. The objective of this study was to compare socioeconomic inequalities in male mortality by oral and pharyngeal cancer in two major cities of Europe and South America.

**Methods:**

The official system of information on mortality provided data on deaths in each city; general censuses informed population data. Age-adjusted death rates by oral and pharyngeal cancer for men were independently assessed for neighbourhoods of Barcelona, Spain, and São Paulo, Brazil, from 1995 to 2003. Uniform methodological criteria instructed the comparative assessment of magnitude, trends and spatial distribution of mortality. General linear models assessed ecologic correlations between death rates and socioeconomic indices (unemployment, schooling levels and the human development index) at the inner-city area level. Results obtained for each city were subsequently compared.

**Results:**

Mortality of men by oral and pharyngeal cancer ranked higher in Barcelona (9.45 yearly deaths per 100,000 male inhabitants) than in Spain and Europe as a whole; rates were on decrease. São Paulo presented a poorer profile, with higher magnitude (11.86) and stationary trend. The appraisal of ecologic correlations indicated an unequal and inequitably distributed burden of disease in both cities, with poorer areas tending to present higher mortality. Barcelona had a larger gradient of mortality than São Paulo, indicating a higher inequality of cancer deaths across its neighbourhoods.

**Conclusion:**

The quantitative monitoring of inequalities in health may contribute to the formulation of redistributive policies aimed at the concurrent promotion of wellbeing and social justice. The assessment of groups experiencing a higher burden of disease can instruct health services to provide additional resources for expanding preventive actions and facilities aimed at early diagnosis, standardized treatments and rehabilitation.

## Background

A large variation of incidence has been reported for oral and pharyngeal cancer worldwide [1]. In Europe, several areas of France and Spain rank among the highest incidence of the disease. In parallel, the city of São Paulo presents the highest incidence of these tumours in Latin America [2]. Variation in cancer incidence reflects differences of risk; although part of this variation may be artifactual, on account of differences in case definition, incomplete ascertainment, and differential access to care and diagnosis. Variation in cancer mortality suffers the influence of other factors; such as heterogeneous access to health care and early diagnosis, patients' comorbidities and the risk of competitive causes of death.

Despite the important role played by genetic factors in carcinogenesis, most cancers are overwhelmingly influenced by environmental and behavioural risk factors. Diet, smoking and drinking patterns, viral infection, occupational and environmental exposures, all factors somehow related to socioeconomic inequalities may account for a major proportion of cancer incidence [3]. In addition to presenting an adverse risk profile, underprivileged social strata make less use of the healthcare system for preventive purposes [4]; have lower access to diagnosis during earlier stages of disease; fewer therapeutic resources; and a poorer prognosis when experiencing cancer [5].

Several studies focusing on the international context have used different analytical schemes to indicate behaviour and geopolitical factors as major determinants of large differences in magnitude, unfavourable trends, and socio-demographic inequalities affecting the mortality by most cancers in general; by mouth and pharynx cancer in particular [6-8]. Although the impact of socioeconomic inequalities on health is hard to reduce, the extent of the mortality gap between population strata supports the hypothesis that at least in part, and in the long term, the epidemiologic profile of cancer mortality can actually be improved by preventive actions and expanded access to currently available technologies of healthcare. Therefore, the assessment of socioeconomic covariates of cancer mortality may be relevant to a full comprehension of distal determinants of the disease, and to appraise opportune interventions [9].

These considerations motivated the current study, which aimed at documenting and comparing socioeconomic inequalities in oral and pharyngeal cancer in two major cities of Europe and South America, which have a large incidence of these tumours. Previous research has examined this theme at the international level by reviewing the literature or comparing broad databases [10,11]. However, the perspective of performing methodologically controlled international comparisons of outcomes related to the experience of disease [12] is a more recent perspective.

This study describes the magnitude, trends and inner-city inequalities of male mortality by oral and pharyngeal cancer in Barcelona and São Paulo, as assessed by uniform methodological criteria. Its main objectives were to quantify inequalities in the burden of disease and to assess the association of death rates with socioeconomic indices at the small-area level.

## Methods

### Design and population

This is an ecological study comparing the mortality by oral and pharyngeal cancer in males living in two large cities: Barcelona, Spain, and São Paulo, Brazil. The system of information on cancer mortality was previously reported as reliable for both cities during the study period [13,14]. The gradient of mortality in each city refers to different death rates estimated for their areas. The current appraisal used the geographic division of both cities into areas with relatively homogeneous socioeconomic features, as previously performed by governmental agencies for administrative purposes.

With 1,512,971 inhabitants in 2000, Barcelona comprises 38 neighbourhoods, which range from 767 to 96,681 inhabitants, 34,026 for the median. São Paulo had 10,434,252 inhabitants in the same year; its administrative division of areas comprises 96 districts, which range from 8,404 to 333,436 inhabitants, 98,705 for the median. Both cities are the core of even larger metropolitan areas.

### Sources of information and covariates

Mortality data were obtained from death certificates gathered by the official system of information on mortality of both countries during the study period, with stratification by gender, age, year, underlying cause and inner-city area of residence. Inclusion criteria refer to deaths by mouth and pharynx cancer in men, as identified by the following codes of the International Classification of Diseases: 140 to 149 (9th revision, used from 1995 to 1999 in Barcelona, and in 1995 in São Paulo), and C00 to C14 (10th revision, used from 1999 to 2003 in Barcelona, and from 1996 to 2003 in São Paulo). Despite entailing different codes, the change from the 9th to the 10th revision of the International Classification of Diseases had no further consequences for the notification of mouth and pharynx cancer deaths [15].

Local censuses performed in 1996 and 2000 (and intercensal estimates) provided population information for the assessment of death rates for both cities. Death rates were adjusted by the direct method, using the age distribution of the new world standard population [16] proposed by the World Health Organization. Figures were expressed in terms of 100,000 inhabitants.

Socioeconomic indices were linked to mortality information at the inner-city area level. The most recent data on socioeconomic status informed the current assessment of small areas in Barcelona (statistical census performed in 2001), and in São Paulo (general census performed in 2000). Indices used as covariates were: (i) the unemployment rate, assessed as the percentage of men aged 15–64 years old who reported being unemployed (Barcelona), and of heads of households who reported having no income at all (São Paulo); (ii) insufficient instruction, referring to persons of the same age group who declared less than four years of formal schooling (Barcelona), and to heads of households (São Paulo); (iii) academic degree, i.e. proportion of persons aged 20 or more years old who reported having completed a University course (Barcelona), and of heads of households reporting 15 years or more of formal schooling (São Paulo); and (iv) the human development index (HDI), a composite measurement aggregating information on three dimensions: income (the unemployment rate), schooling (insufficient instruction and academic degree) and longevity (life expectancy) in 2001 (Barcelona) and 2000 (São Paulo), according to criteria set up by the United Nations Development Program [17]. Table 1 synthesises the assessment of socioeconomic indices.

**Table 1:** Definition of socioeconomic indices assessed in Barcelona, Spain, 2001, and São Paulo, Brazil, 2000, as covariates for oral and pharyngeal cancer mortality in 1995–2003.

**Socioeconomic indices**	**Barcelona**	**São Paulo**
Unemployment rate	Proportion of men aged 15–64 years old who reported being unemployed.	Proportion of heads of households who reported having no income at all.
Insufficient instruction	Proportion of men aged 15–64 years old who reported less than four years of formal schooling.	Proportion of heads of households who reported less than four years of formal schooling.
Academic degree	Proportion of men aged 20 or more years old who reported having completed a University course.	Proportion of heads of households who reported 15 or more years of formal schooling.
Human development index	Composite measurement aggregating information on income (unemployment rate); schooling (insufficient instruction and academic degree) and longevity (life expectancy).	Composite measurement aggregating information on income (unemployment rate); schooling (insufficient instruction and academic degree) and longevity (life expectancy).

### Data analysis

Statistical analyses used the SPSS 8.0 1997. Aiming to provide reliable estimates of mortality, time series assessed yearly death rates for the city as a whole, and the geographic assessment of mortality comprised estimating rates for each district as referred to the whole study period. Mortality rates for each district were subsequently divided by the number of years in the study period, in order to provide figures comparable in magnitude to the overall mortality. Socioeconomic indices were also assessed for each district in both cities. The number of deaths assessed for females and for each anatomic site was not sufficiently high to allow stratification by area of residence.

For both Barcelona and São Paulo, inner-city areas were ordered into a gradient according to the mortality of men by oral and pharyngeal cancer. This gradient allowed the division of areas into terciles with descriptive purposes, and averages for socioeconomic indices were presented for each tercile (low, medium and high mortality), thus indicating the correlation between the outcome and covariates.

Trend estimation used the Prais-Winsten procedure of regression analysis [18], which corrects first-order temporal autocorrelation of residues. Ecological correlations between mortality and socioeconomic indices of areas used the simultaneous autoregressive (SAR) scheme of generalized least squares regression analysis [19], which corrects first-order spatial autocorrelation of residues. Regression models were fitted independently for each city, for the subsequent comparison of outcomes.

## Results

**Figure 1:**
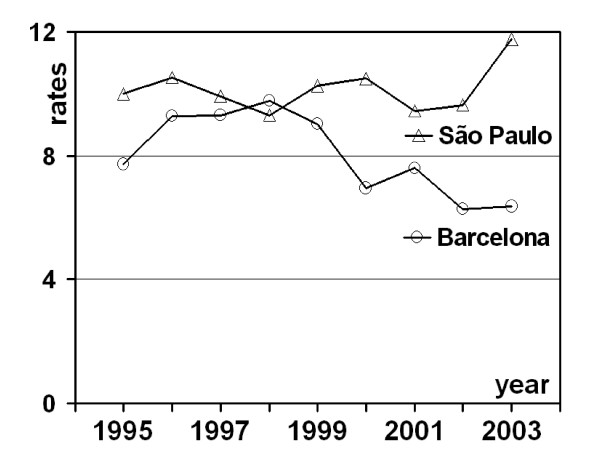
**Age-adjusted mortality (per 100,000 male inhabitants) by oral and pharyngeal cancer in males. **Time series for Barcelona, Spain, and São Paulo, Brazil, 1995–2003.

Male mortality by oral and pharyngeal cancer ranked higher for São Paulo than for Barcelona (table 2). Trend estimation provided a further favourable condition for Barcelona in the comparison with São Paulo, because rates decreased for the former city at the short term (1995 to 2003), and remained stationary for the latter (figure 1). Throughout the study period, 740 men died from the disease in Barcelona, with area-level mortality ranging from 0 to 20.9 deaths per 100,000 inhabitants. The corresponding figures for São Paulo were: 3,211 (overall number of deaths), 3.2 (minimum area-level death rate), and 15.7 (maximum).

**Table 2:** Oral and pharyngeal cancer mortality in Barcelona, Spain, and São Paulo, Brazil, 1995–2003: number of deaths by anatomic site, overall death rate (per year, age adjusted, per 100,000 inhabitants) and trends (rate of yearly increase, 95% confidence interval).

**Male mortality**	**Barcelona**	**São Paulo**
Lips	15 (2.0%)	17 (0.5%)
Tongue	176 (23.8%)	868 (27.0%)
Oral cavity	167 (22.6%)	689 (21.5%)
Salivary glands	26 (3.5%)	99 (3.1%)
Oropharynx	120 (16.2%)	803 (25.0%)
Nasopharynx	75 (10.1%)	151 (4.7%)
Hypopharynx	120 (16.2%)	261 (8.1%)
Ill-defined parts of mouth and pharynx	41 (5.6%)	323 (10.1%)

Total number of deaths	740 (100%)	3211 (100%)

Overall death rate (per year)	8.04	10.23
Rate of yearly increase	-4.18%	+0.91%
95% Confidence Interval	-7.58% to -0.67%	-0.61% to +2.45%
Trend interpretation	decrease	stationary

**Figure 2:**
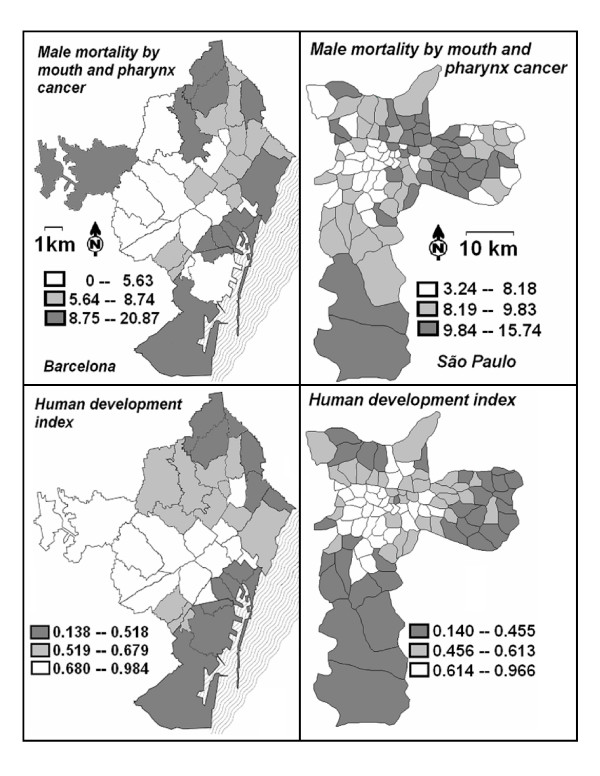
**Age-adjusted mortality (1995–2003, per 100,000 male inhabitants) by oral and pharyngeal cancer in males, and human development index. **Tercile distribution in areas of Barcelona, Spain, and São Paulo, Brazil.

The tercile distribution of area-level rates highlighted the spatial gradient of mortality in both cities (figure 2). Areas presenting the lowest figures (first tercile) mostly occupy prosperous regions of Barcelona and São Paulo; whereas those with higher death rates (second and third terciles) tend to be located in deprived regions of both cities. The ratio between mortality medians for the third and the first tercile ranked 2.45 for Barcelona and 1.99 for São Paulo, indicating a larger gradient of mortality for the former city.

In general, the area-level distribution of mortality overlapped the geographical assessment of socioeconomic indices for both cities. Poorer neighbourhoods of Barcelona mostly occupy its Mediterranean coast and northern region. São Paulo's deprived areas are located at the outermost regions of the city, in addition to a few once prosperous but now impoverished central districts. The distribution of areas by socioeconomic indices broadly matches the distribution of male mortality by oral and pharyngeal cancer in both cities, as indicated in figure 2 and quantitatively assessed in table 3.

**Table 3:** Tercile distribution of the age-adjusted mortality by oral and pharyngeal cancer in males (Barcelona, Spain, and São Paulo, Brazil, 1995–2003): medians for the death rate and socioeconomic indices; area-level correlation coefficient.

**Barcelona**	**1^st ^tercile**	**2^nd ^tercile**	**3^rd ^tercile**	Correlation coefficient*
Age-adjusted death rate	4.78	6.49	11.69	--
Unemployment rate	9.8%	10.7%	12.6%	+0.618 (p < 0.001)
% Low instruction	3.3%	5.1%	9.1%	+0.652 (p < 0.001)
% Academic degree	17.0%	8.5%	7.0%	-0.317 (p = 0.026)
HDI**	0.693	0.622	0.403	-0.515 (p < 0.001)

**São Paulo**	**1^st^tercile**	**2^nd^tercile**	**3^rd ^tercile**	Correlation coefficient*

Age-adjusted death rate	6.10	9.04	12.15	--
Unemployment rate	6.1%	10.8%	8.7%	+0.118 (p = 0.127)
% Low instruction	7.9%	20.2%	17.6%	+0.308 (p = 0.001)
% Academic degree	31.9%	7.8%	7.6%	-0.472 (p < 0.001)
HDI**	0.708	0.479	0.547	-0.348 (p < 0.001)

* Correlation between each socioeconomic index and mortality, at the area level.

** HDI: human development index.

Male mortality by the disease associated with socioeconomic conditions of areas in both cities (table 3). However, goodness-of-fit indicators ranked lower for São Paulo, and the unemployment rate did not significantly correlate with mortality in this city. In addition to presenting a more unequal profile of mortality than São Paulo, Barcelona had higher SAR correlation coefficients for all covariates, which indicates a closer fit between male mortality by oral and pharyngeal cancer and socioeconomic indices.

Areas with poorer socioeconomic standings tended to present higher levels of mortality for males living in Barcelona and São Paulo. In order to appraise the socioeconomic feature of the area-level gradient of mortality, table 4 indicated the proportional increment of mortality, as associated with a 10% increment for each covariate and unadjusted for the remaining modelled conditions.

**Table 4:** Proportional increment or decrement of mortality by oral and pharyngeal cancer associated with a 10% increment of each socioeconomic index, in Barcelona, Spain, and São Paulo, Brazil, 1995–2003.

**Barcelona**	**Increment of mortality**	**95% confidence interval**
Unemployment rate	+15.46%	+8.81% to +22.10%
% Low instruction	+5.29%	+3.22% to +7.37%
% Academic degree	-2.73%	-5.45% to -0.01%
HDI*	-8.24%	-12.88% to -3.61%

**São Paulo**	**Increment of mortality**	**95% confidence interval**

Unemployment rate	+0.83%	-0.61% to +2.27%
% Low instruction	+2.19%	+0.80% to +3.58%
% Academic degree	-1.70%	-2.35% to -1.05%
HDI*	-3.55%	-5.51% to -1.59%

* HDI: human development index.

The grading of inequalities affecting the burden of disease in Barcelona indicated that areas with an unemployment rate 10% higher than the average tended to present a 15.5% increase of male mortality, while the corresponding figure for São Paulo did not differ significantly from zero. Analogously, a 10% higher figure for the index assessing low instruction associated with a 1.5% increment of mortality in São Paulo, and with a significantly higher increase in Barcelona (5.3%). As refers to the proportion of subjects with academic degree, and the human development index, a 10% higher figure associated with higher decrements of mortality for Barcelona than for São Paulo.

Barcelona presented a preferable trend and a lower magnitude of oral and pharyngeal cancer deaths than São Paulo. However, its better overall profile was accompanied by a higher polarization of disease in poorer neighbourhoods of the city. The inner-city gradient of mortality was larger for Barcelona (tables 3 and 4), and deprived areas of this city experienced male mortality by oral and pharyngeal cancer even higher than the poorest areas of São Paulo, thus accounting for a higher inequality in the burden of disease.

## Discussion

This study highlighted two important findings. The first one is the report of elevated mortality in men residing in both cities. This issue represents a major problem for São Paulo, which had higher and levelled-off figures during the study period. The second finding is the identification of mortality as inequitably distributed in both cities, with poorer areas presenting significantly higher death rates. This issue represents a major problem for Barcelona, which had larger inner-city differentials. Notwithstanding, we observe that the smaller size of geographical areas in Barcelona may have contributed to a larger variation of mortality and socioeconomic indices.

Male mortality by oral and pharyngeal cancer decreased in Barcelona at the short term. While comparing recent (1999) with earlier (1983) levels, Barcelona's health authority indicated an overall increase of 39.8% [20]. Although ranking lower than São Paulo, Barcelona presented a high male mortality in terms of the European context. The reassessment of death rates, as adjusted by the standard European population [15], indicated a figure (9.45 yearly deaths per 100,000 inhabitants) higher than those registered for Spain as a whole (8.44 in 2002), the European Union (8.32 in 2003), and European Union members before May 2004 (7.44 in 2002) [21].

The corresponding figure for São Paulo is even higher: 11.86. The disease's incidence and mortality had already been reported as elevated in the Brazilian context, regardless of an estimated underreport of cases in poorer regions of the country [21]. Prior international comparisons considered São Paulo as ranking among the highest mortality by oral and pharyngeal cancer worldwide [11,22,23].

Social inequalities in this outcome had already been reported for Barcelona [13], São Paulo [14], and other contexts [24]. This study adds evidence, by assessing current information on the matter, grading the burden of disease, and comparing the strength of associations between mortality and socioeconomic indices in both cities. The correlation between socioeconomic indices and the gradient of deaths should consider differential incidence among social strata, inequalities in the provision and effectiveness of health services, and the delay in diagnosis and start of treatment.

Regarding the major known risk factors for oral and pharyngeal cancer, smoking [25,26], alcohol abuse and dependence [27,28], and poorer eating habits [29,30] have been reported as more prevalent for deprived population segments in urban areas at the Brazilian and the European context. Deprived social strata have long been recognized as subjected to a higher risk and lower survival of some cancers. Therefore, the continuous monitoring of inequalities in cancer indices may contribute for health services to apply more resources to groups presenting higher needs; thus preventing what Whitehead [31] called "health inequities", i.e. inequalities associated with avoidable, unnecessary and unjust differences in health.

Provision, access and effectiveness of health services are relevant determinants of cancer mortality; and significant inequalities in medical contacts and preventive actions have been reported as a major issue of contemporary societies [4]. The Brazilian and the Spanish national health systems were reformed during the 1980s, when decentralisation and universal access were implemented to expand the free delivery of preventive and therapeutic resources. Despite this common background, universal accession to medical services does not guarantee equity of their effective use, nor homogeneous quality; and inequalities have been reported to affect healthcare in both countries. Being ecological in design, this study could not assess the inner-city association of cancer mortality and access to health services, because there is no correspondence between living areas and the location of medical units, and inhabitants are not restrained to circulate among the city's areas in search for health care.

Recent studies reported that low-income individuals present greater need and lower consumption of medical care, and are less likely to benefit from supplemental private health insurance in Brazil [32,33]. In Barcelona, a less unequal profile for the utilisation of health services was reported [34]; although inequalities remained affecting the distribution of preventive and supplemental services, with deprived social classes presenting a lower prevalence of having a mammography, pap smears, and regular dental services [35]. The report of socioeconomic discrepancies affecting oral and pharyngeal cancer mortality in the context of a more equitable health service (Barcelona) demands further assessments of the potential impact of structural conditions on the determination of health inequalities [36].

The current study used standardized criteria for assessing mortality and socioeconomic indices in the European and the Brazilian context, thus fulfilling requisites of internal validity for comparative analyses. The estimation of death rates gathered official information on population and mortality; the adjustment of death rates used the same standard of population and structure for stratification by age in both cities. Socioeconomic indices also had an analogous assessment, although some heterogeneity remained irreducible due to differences in surveying in each city.

The statistical census in Barcelona specifically inquired about unemployment while the proportion of subjects with no income was taken as a proxy for the unemployment rate in São Paulo; because the socioeconomic form of the Brazilian general census did not include a specific question about unemployment. Moreover, the surveying of socioeconomic standings referred to adults in general, in Barcelona, and exclusively to heads of households in São Paulo. These differences restrained the spatial data analysis, and are acknowledged as a limitation of the study. Anyhow, some collinearity is expected to affect similar assessments of socioeconomic status.

The assessment of distal determinants of mortality may be improved in Brazil if official inquiries and research groups can implement indicators of social deprivation used in European countries. These indicators have elements that enable the measurement of potential workload or pressure on the services of general practitioners [37], or material deprivation [38,39]. Refining those indicators, adapting them to Brazilian conditions could help to orientate future research.

The assessment of correlations between figures aggregated at the area level does not take into account relevant variation of individual socioeconomic characteristics, which may be relevant to the risk of cancer. We observe that the validity of conclusions in ecologic studies comprises the risk of being affected by the ecological fallacy, when observations based on aggregate data are improperly inferred to individual level. In spite of this observation, there is sufficient evidence supporting spatial data analysis as an effective enhancement for studies aiming at improved interventions in public health [40,41].

One further limitation refers to the relatively simple analytical scheme, which does not account for non-linear relationships between mortality and socioeconomic status. In Barcelona, the area-level distribution of death rates suggests an exponential gradient, because the second tercile of neighbourhoods ranked a 36% higher median value than the first tercile, while the third tercile almost doubled the corresponding figure for the second one (table 2). A power function could improve the goodness-of-fit for this assessment; although a more complex mathematical modelling could involve loss in readability and reproducibility of the assessment.

This study assessed socioeconomic conditions as distal determinants of inequalities in oral and pharyngeal cancer mortality among males. The comparison was restricted to two large and important cities that present a large incidence of these tumours. This strategy was exploratory, and comprises an early step of a wider assessment of cancer mortality at the international context. Therefore, the current study is not representative of any broader context; and further studies assessing this complex field are strongly recommended.

## Conclusion

Both Barcelona and São Paulo presented high magnitude and large socioeconomic gradient of male mortality by oral and pharyngeal cancer. In spite of a lower magnitude and a better trend of death rates, Barcelona had a larger socioeconomic gradient of death rates than São Paulo. These findings are indicative of the much that remains to be done to reduce inequalities in the burden of disease, and to improve cancer statistics in both cities.

The acknowledgement of inequalities in health may contribute to the formulation of redistributive policies aimed at the concurrent promotion of wellbeing and social justice. For this aim, health systems demand selective information quantifying inequalities in the experience of disease.

Socioeconomic inequalities of cancer mortality suggest that at least in part, and in the long term, the overall burden of disease can be reduced by primary prevention and structural strategies as control of tobacco and alcohol consumption; by early diagnosis; standardized treatments and rehabilitation. Socially appropriate health programs should encompass universal access and/or the target of additional resources for population segments with higher levels of needs. Such strategies represent the main source of expectation for the reduction of socioeconomic inequalities in health.

## Competing interests

The authors declare that they have no competing interests.

## Authors' contributions

JLFA conceived the study, performed statistical analyses and had the main responsibility for writing the manuscript. CB helped to conceive the study, and advised statistical analyses. GP, AFB and VWF participated in the designing of the study, and contributed in the assessment of results. All authors participated in the drafting and revising of the text.
